# Potassium Hydroxide Extraction of Polyphenols from Olive Leaves: Effect on Color and Acrylamide Formation in Black Ripe Olives

**DOI:** 10.3390/foods13193180

**Published:** 2024-10-07

**Authors:** Mercedes Brenes-Álvarez, Pedro García-García, Eva María Ramírez, Eduardo Medina, Manuel Brenes, Concepción Romero

**Affiliations:** Food Biotechnology Department, Instituto de la Grasa (IG), CSIC, Ctra. Utrera km 1, Building 46, 41013 Seville, Spain; mbrenes@ig.csic.es (M.B.-Á.); pedrog@ig.csic.es (P.G.-G.); evamariaramirez@ig.csic.es (E.M.R.); emedina@ig.csic.es (E.M.); brenes@ig.csic.es (M.B.)

**Keywords:** table olives, acrylamide, potassium hydroxide, hydroxytyrosol, olive leaves extraction

## Abstract

Olive leaves are generated in large quantities in olive oil and table olive factories. This waste is currently used for multiple purposes, one of them being the extraction of bioactive substances, in particular phenolic compounds. The aims of this study were (i) to obtain a new polyphenolic extract from potassium hydroxide (KOH) -treated olive leaves; and (ii) to reduce acrylamide formation in black olives by using this extract. The results showed that olive leaves and leafless branches of the Manzanilla cultivar treated with 10 g/L KOH provide a solution that, concentrated under vacuum, had >6000 mg/L hydroxytyrosol and >2000 mg/L of hydroxytyrosol 4-glucoside. Moreover, the residual material generated after the treatment with KOH could be used for agronomic purposes, due to its high potassium content. The employment of this non-bitter extract during the darkening step of black ripe olive processing then resulted in darker fruits with higher potassium content. Likewise, the addition of the extract into the packing brine reduced the acrylamide formation by up to 32%, although this effect was batch-dependent. KOH olive extract could be useful for the reduction in acrylamide in black ripe olives along with the enrichment of this product in phenolic compounds and potassium.

## 1. Introduction

Olive leaves are generated in large quantities during the pruning of the trees in olive groves and during the cleaning process of the fruits in olive oil and table olive factories. This residue is currently destined to become animal food, burned, ground, and scattered as vegetal cover on the soil, for composting, and many other uses. The extraction of bioactive substances, in particular phenolic compounds, is a field of great interest due to their many recognized applications in foods, cosmetics, and dietary supplement sectors [1,2]. 

The main polyphenol in olive leaves is the secoiridoid oleuropein, which can reach up to 7% in fresh leaves, depending on its concentration on the cultivar, the season, and many other factors [3,4]. Hence, most of the commercial olive leaf extracts are rich in this substance [5], whose bitterness entails a drawback for food application. 

Before the isolation of phenolic compounds, olive leaves are frequently dried using different methodologies at temperatures between 40–120 °C [6,7,8]. Subsequently, these substances are extracted with water or a mixture of water/ethanol [9], applying maceration, microwaves, ultrasound or moderate field techniques [10,11], and they can be further purified by ultrafiltration and nanofiltration [12,13]. The lack of data on the use of the extracted olive leaves, in particular when they contain residual solvent, should be mentioned.

Among the food applications of the olive leaf extract, their use for the reduction in acrylamide formation in black olives has recently attracted much attention [14,15]. The toxic compound acrylamide is formed in black olives during the sterilization stage, and its content ranges between 150–2000 µg/kg in the final product [16]. Martín-Vertedor et al. [14] reported that pure standards of 20 mM oleuropein and tyrosol did not influence the final level of acrylamide in olives, whereas 20 mM hydroxytyrosol and the olive leaf extract meant a reduction in the content of this substance of up to 13% and 38%, respectively. Later, these researchers found that up to a 60% reduction in acrylamide was possible, by adding a mixture of 20 mM hydroxytyrosol and the olive leaf extract, containing 51 mM hydroxytyrosol and 13 mM oleuropein, to the canning brine [15]. Nonetheless, Casado et al. [17] did not detect any significant effects on acrylamide formation in black olives after the addition of 50 mM hydroxytyrosol to the canning brine. This contradictory activity has also been observed in virgin olive oil polyphenols [18,19], which has been attributed to the different composition of the oils. 

The addition of olive leaf extract to the canning brine may also favor a darker color of the black olives [15], which is predictable because the black color of this product is formed as a consequence of the oxidation and polymerization of the olive polyphenols, in particular hydroxytyrosol [20]. With this intention, the reuse of the storage brine of the olives, rich in hydroxytyrosol, during the darkening stage also contributes to obtaining darker fruits [21]. Another benefit of using olive leaf extract and storage brines during the processing of black olives is precisely the enrichment of this product in phenolic antioxidants, particularly hydroxytyrosol [15,21,22]. 

In this study, the method to obtain some olive leaf extract rich in hydroxytyrosol through the alkaline treatment of small branches with potassium hydroxide (KOH) was researched. Additionally, the study explored the impact of this extract on acrylamide formation, color, and phenolic content of black olives.

## 2. Materials and Methods

### 2.1. KOH Treatment of Olive Leaves and Branches

Small tree branches of the Manzanilla cultivar (20–40 cm in length) were picked in the Seville province (Spain) in March. The leaves were separated from the branches, and 70 g were immersed in 140 mL of 10 and 20 g/L KOH solutions at ambient temperature (22–25 °C). The changes in the phenolic compounds in the alkaline solutions were monitored for 24 h. 

Alternatively, the leaves were cut into strips of 2–3 mm in width. Subsequently, 9 g of the olive leaf strips were put inside a 20 mL vial and covered with 10.5 mL of 5 g/L and 10 g/L KOH. The vials were closed and stored at ambient temperature (22–25 °C) for 0.5, 1, 2, 4, 9, and 24 h in duplicate. Around 1 mL of the alkaline solution was taken off the vials using a syringe (1 mL), and the solution was acidified with a drop of phosphoric acid to avoid polyphenol oxidation. The analysis of phenolic compounds was carried out as explained below.

In addition, leafless branches were cut to 60–100 mm lengths, and 90 g were covered with 150 mL of 10 g/L KOH inside a 200 mL jar. After being closed, the jars were stored at ambient temperature (22–25 °C) for 2, 4, 8, 12, and 24 h in duplicate. The analysis of the phenolic compounds in the alkaline solution was carried out as stated above. 

The concentration of KOH used would not release lignin from cell walls [23].

### 2.2. Processing of KOH Olive Leaf Extract (KOLE)

Olive tree branches of 20–40 cm in length and less than 1 cm in diameter of the Manzanilla cultivar were cut into small pieces (<1 cm of leaves and leafless branches) using a shredder MSHP2800d-2 (Mac Allister, Indianapolis, IN, USA). Four kilograms of the cut material were put inside cylindrical stainless equipment (40 cm diameter, 60 cm long) and firmly pressed with a perforated lid. Subsequently, the material was covered with 10 L of 10 g/L KOH and left at ambient temperature (22–25 °C) for 24 h. Then, 5 L of the alkaline solution were concentrated to 20% of the initial volume in a rotavapor R-220 (BUCHI Ibérica S.L.U., Barcelona, Spain), under vacuum at 60 °C. 

### 2.3. Processing of Dried Olive Leaf Extract (DOLE)

The process was based on the methodology reported elsewhere [8]. Leaves of the Manzanilla cultivar were dried using the equipment IRCDi3HP-V (IRconfort Company S.L., Mairena del Aljarafe, Seville, Spain) based on infrared and air convection heating. After drying at 80 °C, leaves were ground using a 6 mm-diameter crushing sieve (ZM200 ultra-centrifugal mill, Retsch GmbH, Haan, Germany), and the phenolic compounds were extracted from 7 g of crushed leaves with 250 mL of boiling water. 

### 2.4. Addition of KOLE during the Darkening Stage of Black Olives

Two different batches (1 and 2) of olive fruits of the Hojiblanca cultivar (1 kg) stored in acidified brine for 6 months were put inside 4 cylindrical oxidation chambers (15 cm diameter, 30 cm long with conical base). Subsequently, they were submerged in one liter of 30 g/L NaOH solution for 3.2 h in order to allow the penetration of the alkali in the pit. After removing the alkali, the olives were covered with one liter of (i) tap water (control), (ii) a mixture of tap water/storage brine (1:1), and (iii) tap water spiked with 128 mL of KOLE. This volume of KOLE was chosen in order to have a similar hydroxytyrosol concentration (850 mg/L) than in the mixture of tap water/storage brine, based on the phenolic composition of both the storage brine and the KOLE (Table 1). After 24 h, all the solutions were discharged and the olives were immersed in fresh tap water and maintained for another 24 h. Acetic acid was added to the suspension olives/liquids during the washings in order to maintain the pH of the liquid in the range of 6.5–8.5 units. To fix the black color formed, the fruits were immersed in a ferrous gluconate solution (1 g/L) for 5 h. Air was bubbled in throughout the whole process. Finally, darkened olives were pitted using an industrial pitting machine (OFM, Dos Hermanas, Spain), bottled (140 g) in A314 jars (Juvasa, Dos Hermanas, Spain), and covered with about 160 mL of a solution containing 32 g/L NaCl and 0.2 g/L ferrous gluconate. Then, the jars were sterilized at 121 °C for 15 min in a Steriflow retort (Madinox, Barcelona, Spain) to reach an accumulated lethality of 15 F_0_. Jars were maintained at ambient temperature for two months before analysis.

### 2.5. Addition of KOLE in Canning Brine of Black Olives

Olives processed as stated above, without the addition of storage brine or KOLE during the first washing (control treatment), were pitted and bottled in A314 jars. The fruits (140 g) were covered with a solution containing 32 g/L NaCl and 0.2 g/L ferrous gluconate (control) or the brine spiked with KOLE to reach 1000 mg/L hydroxytyrosol (H1) and 2000 mg/L hydroxytyrosol (H2). Then, the jars were sterilized as detailed above. 

Another 6 batches of pitted black olives obtained from 6 different table olive companies were bottled in A314 jars to check the effect of KOLE on acrylamide formation. The fruits (140 g) were covered with a solution containing 32 g/L NaCl and 0.2 g/L ferrous gluconate (control) or the brine spiked with KOLE to reach 1000 mg/L hydroxytyrosol. Then, the jars were sterilized to reach an accumulated lethality of 15 F_0_. 

### 2.6. Effect of DOLE and Storage Brine on Acrylamide Formation in Black Olives

Pitted black olives from a table olive company with a flesh pH of around 7 units were bottled in A314 jars containing 140 g of fruit and 160 mL brine. The cover brine had 32 g/L NaCl and 0.2 g/L ferrous gluconate (control) or it was spiked with (i) storage brine, (i) nano-filtered storage brine, and (iii) DOLE. The initial pH of the storage brine (4.0 units) was adjusted to 7 units with 2M NaOH prior to its addition to the packing brine. Likewise, the storage brine was nanofiltered through an Ultracel 3KDa cut-off membrane (Millipore Corp., Billerica, MA, USA) into an Amicon 50 mL stirred cell (Merck Group, Darmstadt, Germany) under nitrogen pressure. Similarly, this was carried out with the storage brine, and the pH of the nano-filtered storage brine was adjusted to 7 units with 2M NaOH. DOLE was added to the cover brine to reach 2300 mg/L oleuropein, which was the major phenolic compound in this extract (95% total polyphenols) [8]. After bottling, the jars were sterilized to reach an accumulated lethality of 15 F_0_.

### 2.7. Analysis of Phenolic Compounds

Phenolic compounds were analyzed according to the methodology reported elsewhere [4]. Briefly, small pieces of pulp (<0.5 g) were cut from 20 olives up to a total of 10 g that were introduced into a solution of 30 mL of DMSO. The mixture was crushed with an Ultra-Turrax homogenizer and, after 30 min of resting contact, it was centrifuged at 6000 g for 5 min, and 0.25 mL of the supernatant was diluted with 0.5 mL of DMSO plus 0.25 mL of 0.2 mM syringic acid (internal standard). The mixture was filtered through a 0.22 µm pore-size nylon filter, and an aliquot (20 µL) was injected into the HPLC chromatographic system, which consists of a Waters 717 plus autosampler, a Waters 600E pump, a Waters column heater module, and a Waters 996 diode array detector (Waters Inc., Mildford, MA, USA). Separation and quantification of each compound was carried out using a Spherisorb ODS-2 (5 μm, 25 × 4.6 mm i.d., Waters Inc.) column, an elution gradient with acidified water and methanol, a flow rate of 1 mL/min and a temperature of 35 °C. Chromatograms were recorded at 280 nm.

Only 2 g of small pieces of leaves were mixed with 30 mL DMSO for the analysis of phenolic compounds in fresh olive leaves. 

The analysis of these substances in the liquids was completed by mixing 0.25 mL of the liquid, 0.5 mL of deionized water, and 0.25 mL of the internal standard (2 mM syringic acid). The mixture (20 µL) was filtered and injected into the chromatograph.

### 2.8. Determination of Olive Color

The color of the olives was measured using a ColorFlex EZ spectrophotometer (HunterLab, Reston, Virginia, USA), equipped with computer software to calculate the CIE L* (lightness), a* (redness), and b* (yellowness) parameters. Interference by stray light was minimized by covering samples with a box that had a matte black interior. The datum from each measurement is the average of 10 olives. To illustrate the color changes between the control sample and samples after treatments, total color variance (ΔE) values were calculated by using the formula:

ΔE = [(ΔL*)^2^ + (Δa*)^2^ + (Δb*)^2^]^1/2^ (1)

where ΔL*, Δa*, Δb* are the differences of these values between the control sample and the samples after treatment. 

### 2.9. Analysis of Acrylamide

Acrylamide in olive pulp was analyzed according to the methodology reported elsewhere [24]. Olives were pitted and then homogenized in a blender, and a portion of 5 g was mixed with 10 mL of ultrapure water and spiked with 100 µL of the internal standard, (^13^C_3_)acrylamide (10 mg/L). The mixture was homogenized for 2 min with an Ultra-Turrax homogenizer and then it was filtered through a filter paper (Whatman n°1440–110). All extracts were finally filtered through a 0.22 μm pore-size nylon filter, and an aliquot (20 μL) was injected into the liquid chromatograph (HPLC).

The quantification of the acrylamide compound was performed using an HPLC-MS system that consisted of a Waters 2695 Alliance with a pump, column heater, and autosampler modules. Detection was carried out with a mass single-quadrupole detector (QDa, Waters, USA). The QDa mass detector was operated in positive mode (ESI+), with the capillary voltage set to 0.8 kV and the cone voltage set to 15 V; nitrogen was used as a nebulizer gas, maintaining the desolvation temperature at 600 °C. A Spherisorb ODS-2 (5 μm, 25 cm × 4.6 mm i.d., Waters Inc.) column was used, and separation was achieved using an isocratic system with a composition of 100% water acidified with 0.6 g/L of formic acid. Later, the column was washed with 100% methanol, and the initial conditions were restored. The total analysis time for HPLC-MS employed was 44 min. The flow rate was 0.4 mL/min, and the column was kept at 35 °C. The mass spectra of acrylamide displayed major signals at m/z 75 and (^13^C_3_)acrylamide at m/z 72. The analyses were carried out in triplicate.

### 2.10. Analysis of Potassium

One gram of olive paste or 1 mL of liquid was digested in DigiPREP equipment (Quebec, Canada) with 25 mL of 14 M HNO_3_ under reflux at 120 °C for 8 h. The mixture was left at ambient temperature, and then 5 mL of HClO_4_/HNO_3_ (4:1, *v*:*v*) was added. Subsequently, the HNO_3_ was evaporated at 140 °C for 3 h under open-air conditions. Finally, the solution was put into a 25 mL graduated flask which was filled with deionized water. Potassium was determined by flame photometry (Sherwood Model 410, Cambridge, England). The apparatus was calibrated with K atomic absorption standard solution (Aldrich Chem. Comp, Teutonia Avenue Milwaukee, Wisconsin 53209 USA).

### 2.11. Statistical Analysis

Statistical analyses were performed using Statistica 8.0 software (StatSoft Inc., 2300 E 14th St Tulsa OK Oklahoma United States 74104, USA). One-way analysis of variance, ANOVA (Duncan’s test), was used to compare mean values with a significance level of 95%.

## 3. Results and Discussion

### 3.1. KOH Treatment of Olive Leaves and Branches

**Figure 1:**
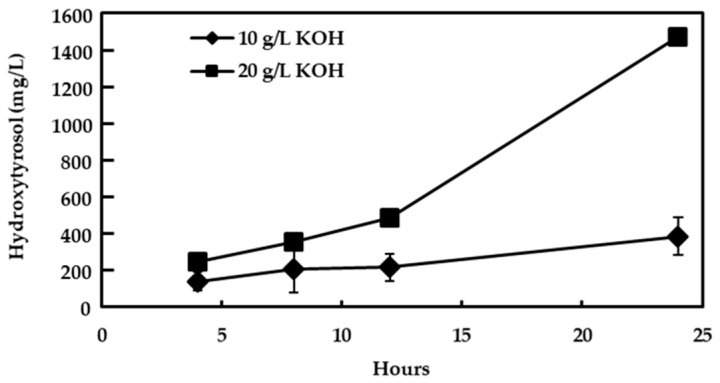
Evolution of hydroxytyrosol in the potassium hydroxide (KOH) solutions of whole olive leaves. Bars mean standard deviation of duplicates.

Oleuropein was the major phenolic compound in the fresh olive leaves of the Manzanilla cultivar, as high as 77.1 g/kg, followed by those of hydroxytyrosol (0.9 g/kg) and verbascoside (0.8 g/kg), as expected from previous studies [4]. When the leaves were immersed in KOH solution, two phenomena could occur, as follows: (i) the diffusion of the phenolic compounds from the leaves to the alkaline solution; and (ii) their hydrolysis, mainly oleuropein. However, the high concentration of triterpenic acids, in particular oleanolic acid, in the leaf surface constitutes a barrier against the diffusion of substances [3,25]. Preliminary assays indicated that a concentration of KOH lower than 10% did not achieve the diffusion of the polyphenols from the leaves to the alkaline solution in hours. The concentration of hydroxytyrosol in the 10 g/L KOH solution of the non-cut leaves was deficient, even after 24 h of contact (Figure 1). With the highest KOH concentration tested (20 g/L), the level of hydroxytyrosol in the liquid was around 1500 mg/L after 24 h of contact, yet it could be increased with prolonged time. It must be noted that only hydroxytyrosol and small quantities of caffeic and p-coumaric acids were detected in these alkaline solutions. Due to the high strength of the alkali and time of contact needed to achieve a rather high concentration of hydroxytyrosol, the alkali treatment of the cut leaves was carried out.

**Figure 2:**
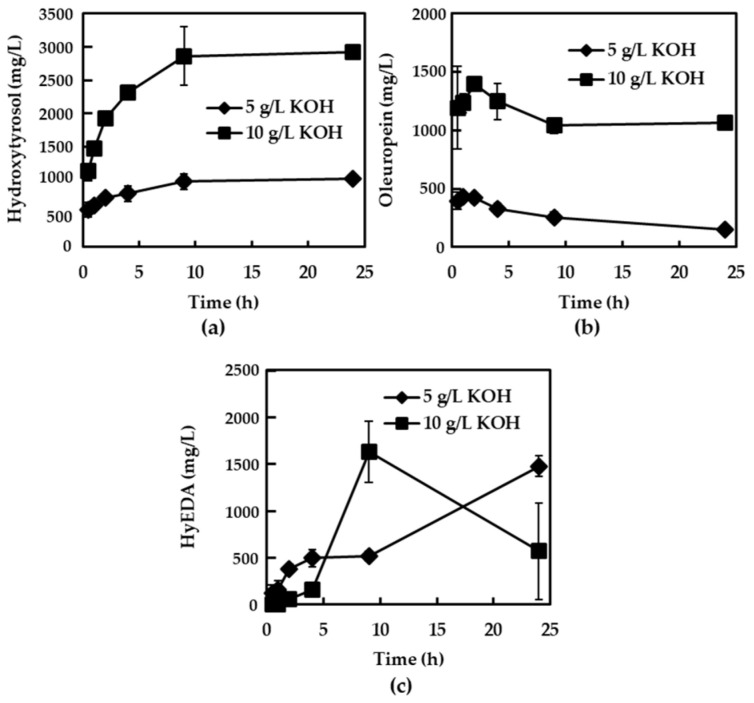
Evolution of phenolic compounds in the KOH solutions of chopped olive leaves. (**a**) Hydroxytyrosol; (**b**) oleuropein; (**c**) dialdehydic form of elenolic acid linked to hydroxytyrosol (HyEDA). Bars mean standard deviation of duplicates.

Under these conditions, a continuous enrichment of the 10 g/L KOH solution in hydroxytyrosol (Figure 2a)—reaching around 2800 mg/L during the first 10 h and remaining constant onwards—was observed. By contrast, the level of hydroxytyrosol obtained using the low alkali strength (5 g/L) along with a low concentration of oleuropein (Figure 2b) was only around 1000 mg/L, which means that this weak alkali strength achieved neither a high rate of oleuropein hydrolysis nor a high diffusion yield of this substance from the leaves. In fact, the content of oleuropein in the 10 g/L KOH solution rapidly increased during the first hour and slightly decreased past that point, with a final concentration of around 1000 mg/L. The detection of the dialdehydic form of elenolic acid linked to hydroxytyrosol (HyEDA) (Figure 2c) in the alkaline solutions is worthy of mention, because this substance is formed via the enzymatic action of β-glucosidase on the oleuropein molecule [26]. During the cutting of the leaves, the enzyme can be released, which allows for the transformation of the secoiridoid into HyEDA [27], its concentration increasing with time in the alkaline solutions, although it also decreased after 10 h in the 10 g/L KOH because of the high pH. Overall, the content of hydroxytyrosol in the potassium solutions could come from the alkaline hydrolysis of oleuropein and HyEDA, and a small quantity from the alkali transformation of verbascoside, which was not detected in these final liquids.

**Figure 3:**
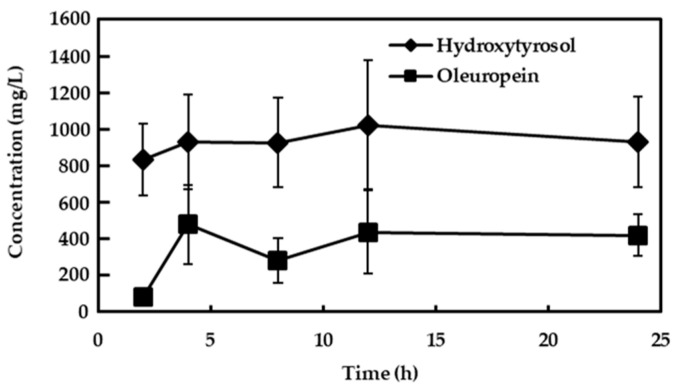
Evolution of hydroxytyrosol and oleuropein in the 10 g/L KOH solution of chopped olive branches. Bars mean standard deviation of duplicates.

Small pieces of leafless branches were also treated with KOH, and results are depicted in Figure 3. In this case, the diffusion and transformation of phenolic compounds were very rapid, as opposed to that which occurred with the leaves. It must be noted that a high concentration of triterpenic acids occurs on the surface of the olive leaves and constitutes a barrier against compound diffusion, which is not the case of the olive branches [28]. In just one hour, a concentration of hydroxytyrosol of around 900 mg/L in the 10 g/L KOH solution was found, which remained almost constant for 24 h. Moreover, a concentration of around 400 mg/L of oleuropein was also detected throughout the 24 h treatment. It must be stressed that the major phenolic compound in the leafless branches was oleuropein (35,593 mg/kg), followed by hydroxytyrosol (1813 mg/L) and ligustroside (243 mg/L), which is in agreement with previous data on the phenolic content in olive branches [28].

### 3.2. Addition of KOLE during the Darkening Stage of Black Olives

The effect of the addition of KOLE in the first washing of the darkening process of black ripe olives on the color of the final product is shown in Table 2.

For batch 1, olives elaborated with KOLE had a significantly darker color (<L* value), than the control, but this effect was not statistically significant for batch 2. Moreover, the addition of storage brine gave rise to significantly darker olives than the control, regardless of the batch, as previously reported [21]. In both cases, the washing solution was enriched in the o-diphenol hydroxytyrosol (Table 1), reported as the main polyphenol involved in the formation of the black color via its oxidation and polymerization under the alkaline conditions [20]. After the first washing, olives are subjected to a new washing step, followed by immersion in the iron fixation solution, and finally canning in a new brine; so the final product was not enriched largely in polyphenols due to the employment of KOLE during the first washing step (Table 2). It must be noted that probably most of the hydroxytyrosol added was oxidized during the washing period, because of the alkaline environment, and it did not penetrate into the olive flesh. By contrast, canned olives from batch 2 previously washed with KOLE had a significantly higher content of potassium (230 mg/kg) than the control (84 mg/kg), which could be expected from the high concentration of this metal added to the first washing solution from KOLE (2420 mg/L) and losses during the subsequent stages. Recently, the elaboration of black ripe olives with KOH instead of NaOH has been proposed, to increase the content of this cation in the final product, and it has other beneficial effects, such as the use of the wastewaters generated for agronomic purposes [29].

### 3.3. Addition of KOLE in Canning Brine of Black Olives

As stated above, the effect of the use of KOLE on the color during the darkening process of black ripe olives was low, so the direct addition of this extract in the canning brine was tested. The addition of KOLE in the canning brine did not have a significant effect on the color of the sterilized product (Table 3). By contrast, it significantly increased the concentration of phenolic compounds in the pulp of the olives, in particular, hydroxytyrosol derivatives, because it seems that oxidation of the o-diphenol hydroxytyrosol occurred—to some extent—during the canning and sterilization stages under neutral pH.

Obviously, the bitter secoiridoid oleuropein was not detected in the canned product. It must be highlighted that black ripe olives are those table olives with the lowest content in these valuable substances [30], so studies have been carried out to enrich this product with polyphenols obtained from several streams generated during the processing of table olives [22].

Another benefit of the addition of olive leaf extract to the canning brine of black ripe olives could be the reduction by up to 38% of the acrylamide formation during the sterilization step [14,15]. With this aim, KOLE was added to the packing brine of six different olive batches, and the results are presented in Table 4. The concentration of acrylamide in black ripe olives was significantly reduced in only two of the six batches tested (22 and 32% reduction). However, the addition of KOLE to the packing brine of four batches of black ripe olives did not significantly manage to inhibit the formation of acrylamide during the sterilization step. Previous olive leaf extracts tested for acrylamide reduction were rich in oleuropein [14,15], similar to most of the commercial olive leaf extracts [31]. By contrast, in this study the main polyphenol in KOLE was hydroxytyrosol, followed by its derivative hydroxytyrosol 4-glucoside, and a lower content of oleuropein (Table 1). It must be highlighted that standards of oleuropein (20 mM) and hydroxytyrosol (50 mM) did not show any effect on acrylamide reduction in black ripe olives [15,17], as opposed to olive leaf extracts rich in oleuropein [14,15]. Therefore, the next assay consisted in testing some olive leaf extract rich in this substance (DOLE).

### 3.4. Effect of DOLE and Storage Brine on Acrylamide Formation in Black Olives

A reduction of 17% in acrylamide formation in black ripe olives was reached by adding DOLE to the packing brine before the sterilization step (Table 5). Although it is not a very high reduction, it could encourage the carrying out of new assays. However, it is noteworthy that the concentration of oleuropein added to the packing brine from DOLE was 2300 mg/L, and it is supposed that the final product contained 814 mg/kg of this bitter substance. Consequently, the bitterness of DOLE could be a problem for its use, as opposed to KOLE, because of the low level of the bitter compound oleuropein in the latter extract. 

Another solution rich in hydroxytyrosol that has been proposed to enrich black ripe olives in this phenolic compound is brine, for the storage of the olives before the darkening stage [22]. Surprisingly, this storage in brine doubled the level of acrylamide in olives, despite its high concentration in hydroxytyrosol (Table 5). Acrylamide formation in most heated foods (>110 °C) occurs mainly from the precursor asparagine and reducing sugars during the Maillard reaction, but this mechanism has been ruled out for black ripe olives [16,32,33], and alternatively, the involvement of peptides smaller than 10 kDa has been suggested for said product [34].

The storage brine was nano-filtered through 3 kDa, and the acrylamide level of the olives was similar to that found using non-filtered brine. Hence, the precursors of the reaction must be smaller than 3 kDa, and the synthesis of the toxic compound was not inhibited by the hydroxytyrosol added. Obviously, the concentration of hydroxytyrosol in the final product significantly increased (571 mg/kg) in comparison with the control (48 mg/kg), but in parallel, the level of acrylamide increased, which represents a big drawback for the reuse of these storage solutions during the canning of olives. 

With respect to the color, neither the employment of the storage brine nor DOLE in the packing brine had a significant effect on this important quality characteristic of olives (Table 5).

## 4. Conclusions

First, olive leaf extract rich in hydroxytyrosol can be obtained from the KOH treatment of olive branches, and the residual material after the treatment with KOH is rich in potassium, so it can be used as fertilizer for agronomic purposes. 

Secondly, the application of the alkaline olive leaf extract during several stages of the elaboration of black ripe olives, darkening, and packing was also assessed. During darkening, this extract was added to the first washing solution, and it gave rise to slightly darker olives with a higher content of potassium. However, its addition to the packing brine did not affect the color of the olives, although (i) it enriched them in hydroxytyrosol and (ii) achieved an acrylamide reduction of up to 32% depending on the olive batch. Likewise, the olive leaf extract water showed a 17% reduction in the acrylamide formation, but it resulted in a very high content of bitter oleuropein in the olives, which represents a drawback for its use during the elaboration of black ripe olives. Interestingly, the addition of the unfiltered and nano-filtered storage brine to the packing solution doubled the amount of acrylamide in the final product, revealing that precursors of acrylamide formation with molecular weights lower than 3 kDa were present in the storage brine. 

The results of this work revealed that the olive leaf extract obtained from the KOH treatment of small olive branches could be added to the packing solutions of black ripe olives for a reduction in acrylamide formation and enrichment of the product in the non-bitter hydroxytyrosol.

## Figures and Tables

**Table 1:** Phenolic compounds content (mg/L) in the storage brine of olives prior to the darkening stage and in the potassium hydroxide (KOH) olive leaf extract (KOLE).

Phenolic Compound	Storage Brine	KOLE
Hydroxytyrosol	1672 ± 20	6551 ± 123
Hydroxytyrosol 4-glucoside	Not detected	2098 ± 19
Oleuropein	77 ± 3	1235 ± 28
HyEDA ^1^	Not detected	566 ± 11
Tyrosol	166 ± 13	117 ± 7

^1^ Dialdehydic form of elenolic acid linked to hydroxytyrosol. Results are expressed as mean ± standard deviation of duplicates.

**Table 2:** Effect of adding a mixture of storage brine/tap water (1:1) or KOH olive leaf extract (KOLE) during the first washing step of black ripe olive processing on the color, phenolic compounds and potassium of the final product. See Table 1 for initial phenolic composition of solutions.

	Color Parameters				Total Polyphenols (mg/kg)	K (mg/kg)
	L*	a*	b*	ΔE ^1^		
*Batch 1*						
Control	21.3a ^2^	3.6a	3.1a		na	na
Mixture brine	19.3b	3.2a	2.0a	2.4	na	na
KOLE	19.0b	3.2a	2.1a	2.6	na	na
*Batch 2*						
Control	20.5a	3.4a	3.4a		72b	84a
Mixture brine	19.2b	3.5a	2.8b	1.0	102a	118a
KOLE	19.9a	3.7a	3.5a	0.6	74b	230b

^1^ Total color variance (ΔE), ΔE = [(ΔL*)^2^ + (Δa*)^2^ + (Δb*)^2^]^1/2^. ^2^Column values followed by the same letter for each batch do not differ at the 5% level of significance according to Duncan’s multiple range test; na, not analyzed.

**Table 3:** Effect of adding KOH olive leaf extract in the packing brine on color and phenolic compounds content of black ripe olives.

	Control	1000 mg/L of Hydroxytyrosol	2000 mg/L of Hydroxytyrosol
*Color*			
L*	20.5a ^1^	20.2a	20.4a
a*	3.4a	3.9a	3.7a
b*	3.4a	3.3a	3.2a
ΔE ^2^		0.5	0.4
*Phenolic compounds (mg/kg)*			
Hydroxytyrosol	29b	160a	159a
Hydroxytyrosol glycol	nd	40b	117a
Hydroxytyrosol 4-glucoside	5c	70b	137a
Tyrosol	15b	24a	23a
Luteolin 7-glucoside	15b	17b	22a
Others	7b	6b	26a
Total polyphenols	71c	317b	484a

^1^ Raw values followed by the same letter do not differ at the 5% level of significance according to Duncan´s multiple range test. nd, not detected. Others mean the sum of vanillic acid, p-coumaric acid, hydroxytyrosol acetate and verbascoside. ^2^ Total color variance (ΔE), ΔE = [(ΔL*)^2^ + (Δa*)^2^ + (Δb*)^2^]^1/2^.

**Table 4:** Effect of adding KOH olive leaf extract to the packing brine on the acrylamide content (µg/kg) of black ripe olives.

Batch	Control	KOLE	Difference (%)
1	226a ^1^	154b	−32
2	355a	276b	−22
3	247a	236a	−4
4	187a	167a	−11
5	428a	402a	−6
6	273a	291a	+6

^1^ Row values followed by the same letter for each batch do not differ at the 5% level of significance according to Duncan´s multiple range test.

**Table 5:** Color, polyphenols and acrylamide content of black ripe olives canned with raw storage brine, nano-filtered storage brine and dried olive leaf extract (DOLE).

	Control	Storage Brine	Nano-Filtered Brine	DOLE
*Acrylamide (µg/kg)*	273b ^1^	582a	529a	226c
*Changes of acrylamide (%)*		+113	+94	−17
*Color parameters*				
L*	18.1a	18.7a	18.6a	17.9a
a*	0.6a	0.3a	0.5a	0.4a
b*	−0.3a	−0.5a	−0.2a	−0.3a
*Polyphenols (mg/kg)*				
Hydroxytyrosol	48b	571a	572a	68b
Hydroxytyrosol 4-glucoside	34b	nd	nd	47a
Tyrosol	20b	64a	57a	19b
Hydroxytyrosol acetate	12b	28a	26a	15b
Oleuropein	nd	nd	nd	814a

^1^ Raw values followed by the same letter do not differ at the 5% level of significance according to Duncan´s multiple range test. nd, not detected.

## Data Availability

The original contributions presented in the study are included in the article, further inquiries can be directed to the corresponding author.
